# The mechanisms of white matter injury and immune system crosstalk in promoting the progression of Parkinson’s disease: a narrative review

**DOI:** 10.3389/fnagi.2024.1345918

**Published:** 2024-05-28

**Authors:** Wen Ma, Yifan Geng, Youhan Liu, Huixin Pan, Qinglu Wang, Yaohua Zhang, Liping Wang

**Affiliations:** ^1^Graduate School of Education, Shandong Sport University, Jinan, Shandong, China; ^2^Xuzhou Clinical School, Xuzhou Medical University, Xuzhou, Jiangsu, China; ^3^Key Laboratory of Biomedical Engineering & Technology of Shandong High School, Qilu Medical University, Zibo, China

**Keywords:** Parkinson’s disease, white matter damage, immune modulation, microglia, oligodendrocytes, T cells

## Abstract

Parkinson’s disease (PD) is neurodegenerative disease in middle-aged and elderly people with some pathological mechanisms including immune disorder, neuroinflammation, white matter injury and abnormal aggregation of alpha-synuclein, etc. New research suggests that white matter injury may be important in the development of PD, but how inflammation, the immune system, and white matter damage interact to harm dopamine neurons is not yet understood. Therefore, it is particularly important to delve into the crosstalk between immune cells in the central and peripheral nervous system based on the study of white matter damage in PD. This crosstalk could not only exacerbate the pathological process of PD but may also reveal new therapeutic targets. By understanding how immune cells penetrate through the blood–brain barrier and activate inflammatory responses within the central nervous system, we can better grasp the impact of structural destruction of white matter in PD and explore how this process can be modulated to mitigate or combat disease progression. Microglia, astrocytes, oligodendrocytes and peripheral immune cells (especially T cells) play a central role in its pathological process where these immune cells produce and respond to pro-inflammatory cytokines such as tumor necrosis factor (TNF-α), interleukin-1β(IL-1β) and interleukin-6(IL-6), and white matter injury causes microglia to become pro-inflammatory and release inflammatory mediators, which attract more immune cells to the damaged area, increasing the inflammatory response. Moreover, white matter damage also causes dysfunction of blood–brain barrier, allows peripheral immune cells and inflammatory factors to invade the brain further, and enhances microglia activation forming a vicious circle that intensifies neuroinflammation. And these factors collectively promote the neuroinflammatory environment and neurodegeneration changes of PD. Overall, these findings not only deepen our understanding of the complexity of PD, but also provide new targets for the development of therapeutic strategies focused on inflammation and immune regulation mechanisms. In summary, this review provided the theoretical basis for clarifying the pathogenesis of PD, summarized the association between white matter damage and the immune cells in the central and peripheral nervous systems, and then emphasized their potential specific mechanisms of achieving crosstalk with further aggravating the pathological process of PD.

## 1 Introduction

Parkinson’s disease (PD), also known as “shaking palsy,” is a common degenerative neurological disease in middle-aged and elderly populations (Tolosa et al., 2021). Its primary pathological features include a reduction in dopamine levels in the brain, a significant degeneration and loss of dopaminergic neurons in the substantia nigra, and the presence of Lewy bodies in the cytoplasm of the remaining neurons (Dauer and Przedborski, 2003; Cacabelos, 2017). Clinically, it is characterized mainly by resting tremor, bradykinesia, muscle rigidity, and postural and balance impairments, along with significant progressive neurodegenerative symptoms such as anxiety, depression, sensory disturbances, and cognitive impairments (Jankovic, 2008; Cacabelos, 2017). Currently, the main treatment methods for PD include pharmacotherapy and surgical interventions, such as Levodopa, dopamine receptor agonists (DAs), deep brain stimulation, and pallidotomy. Patients with PD have significant on–off phenomena, such as levodopa treatment resistance manifesting as an on–off effect, where patients experience fluctuations in motor function with continued use of the drug. These fluctuations may lead to periods of improved motor performance (on periods) followed by a sudden and unpredictable loss of efficacy (off periods). This phenomenon is thought to be the result of a complex interaction between the pharmacokinetics of levodopa, disease progression, and dopamine receptor sensitivity in the basal ganglia (Nutt, 1987). However, as the disease progresses, long-term use of medications can lead to side effects such as dyskinesia, and the surgical treatments available have short-term effects and significant side effects (Ramirez-Zamora and Molho, 2014).

In recent years, an increasing number of studies have come to view PD as a multisystem disorder characterized by significant neuroinflammation and immune dysfunction. This has led to a growing interest in exploring the relationship between PD and the immune system, uncovering the potential crucial role the immune system may play in the development of PD (Witoelar et al., 2017; Tan et al., 2020; Olson et al., 2021; Tansey et al., 2022). Historically, the central nervous system (CNS) was considered an “immunologically privileged” site, partly due to the presence of the blood–brain barrier (BBB), which restricts the entry of immune cells into the brain (Engelhardt et al., 2017). Additionally, neurons within the brain typically do not express or only express at low levels those major histocompatibility complex (MHC) molecules that could initiate an immune response. However, recent studies have revealed a more complex scenario where, under certain conditions, neurons can express MHC molecules and the immune system, along with neuroinflammatory factors, can become active within the brain. This activity can contribute to the development of neurodegenerative diseases like PD (Tansey et al., 2022). Thus, the precise mechanisms by which the immune system and neuroinflammatory factors interact and influence the pathogenesis of PD remain unclear.

The role of immune senescence, often overlooked in neurodegenerative disease research, is characterized by immune deficiencies and inflammation that increase with age (Tansey et al., 2022). In the early stages of PD, immune cells, particularly microglia located in the substantia nigra and striatum, play a crucial role in the disease’s progression (Gerhard et al., 2006). Chronic inflammation in the brains of PD patients may stem from the abnormal activation of these microglia, which are meant to clear dead or damaged cells but instead lead to neuronal damage in PD. Activation of microglia may lead to neuroinflammation and neuronal apoptosis by generating excessive cytokines and oxidative stressors. These cytokines and oxidative stressors can disrupt normal neuronal function, damage neuronal structure and metabolism, and even lead to neuronal death. For instance, microglial activation induces the production of pro-inflammatory cytokines and chemokines via Toll-like receptors (TLR) and interferon-γ (IFN-γ) signaling, upregulates expression of NADPH oxidase and inducible nitric oxide synthase, releases reactive oxygen species (ROS) and nitric oxide (NO), and produces molecules such as matrix metallopeptidase 12 (MMP12), ultimately resulting in inflammation and neurotoxicity (Colonna and Butovsky, 2017).

The role of inflammatory factors, especially the overexpression of TNF-α, is significant, potentially leading to inflammatory damage and suppression of the immune system (Kox et al., 2014). Studies in the PD mouse have shown that inhibiting TNF-α can significantly reduce neuroinflammation and the loss of dopaminergic neurons, while knocking out the TNF-α receptor offers protection in PD model mice (Harms et al., 2011), suggesting TNF-α plays a role in the early stages of PD by promoting inflammatory responses and neuronal damage. When microglia are activated, they produce pro-inflammatory factors, leading not only to degeneration and necrosis of neurons but potentially initiating an immune response against dopaminergic neurons by acting as antigen-presenting cells, particularly through the increased presence of CD4^+^ and CD8^+^ T cells, emphasizing the role of adaptive immunity in PD progression (Tansey et al., 2022).

The connection between core pathological markers, such as α-synuclein (α-Syn), and inflammation and immune dysfunction has been a focus of many studies. For instance, it was found that CD4^+^ T cells could recognize α-Syn (Sulzer et al., 2017), promoting the spread of inflammation, further highlighting the immune system’s role in PD pathogenesis. Previous research has mainly focused on the substantia nigra in the midbrain, a key feature of PD due to the degenerative changes in dopaminergic neurons in this region. However, recent studies have started to pay attention to other areas of the brain, especially white matter, finding that damage to white matter might also play a significant role in PD development (Oveisgharan et al., 2021). White matter contains numerous nerve fiber bundles responsible for transmitting information between different brain regions (Bohnen and Albin, 2011). α-Syn accumulates abnormally and is transported along axons to the white matter of the brain, where a variety of neurotransmitters, such as glutamatergic, cholinergic, and dopaminergic, released by axons and glia in the white matter of the brain, can cause neuronal deformation and death if the tissues are ischemic and hypoxic. In PD patients, white matter damage could obstruct neural signal transmission, affecting coordination and function across the brains (Bohnen and Albin, 2011), potentially related to cognitive decline, motor function impairment, and worsening of other PD symptom (Bohnen and Albin, 2011; de Laat et al., 2012). Moreover, white matter damage might be linked to the observed inflammation and immune dysfunction in PD (Bohnen and Albin, 2011), with microglia and astrocytes within white matter playing key roles in the inflammatory response (Durrenberger et al., 2012). These cells may be activated during PD pathological process, exacerbating the condition.

In summary, research on PD is evolving from a focus solely on the damage to dopaminergic neurons in the substantia nigra to a more comprehensive understanding of damage in different brain areas, including white matter, and how these damages interact with inflammation, the immune system, and core pathological markers of PD such as the abnormal aggregation of α-Syn. This holistic perspective is crucial for a better understanding of the pathological mechanisms of PD and for the development of new therapeutic strategies.

## 2 White matter damage and Parkinson’s disease

### 2.1 Overview of white matter damage

White matter, a crucial component of the central nervous system, primarily consists of nerve fibers and their surrounding myelin sheaths, which give white matter its characteristic white appearance (Yang et al., 2023). These structures play a vital role in the brain, including facilitating rapid information transmission, maintaining the stability of neural networks, and supporting cognitive functions (Cainelli et al., 2020). Damage to the nerve cells or myelin within white matter, known as white matter damage, encompasses a range of diseases involving impaired white matter integrity and demyelination (Yan et al., 2021). White matter is particularly susceptible to insufficient blood supply in the subcortical white matter areas, leading to hypoxia and ischemia, which can trigger inflammatory responses and BBB breakdown. This disruption exacerbates the accumulation of toxins within the brain, damages vascular endothelial cells, and further reduces white matter blood flow, creating a vicious cycle that aggravates the pathology (Egashira et al., 2015; Toyota et al., 2019). A large-scale genome-wide association (GWAS) revealed the genetic architecture of white matter microstructure and its overlap with cognitive and mental health traits, suggesting a genetic basis for white matter variations that may affect brain function and its immune interactions (Zhao et al., 2021).

**Figure 1:**
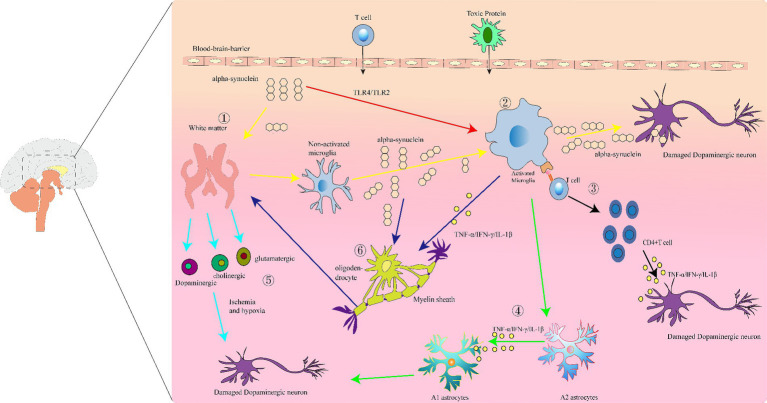
Immunoinflammatory manifestations of Parkinson’s disease (PD): (1) white matter damage. Abnormal aggregation of α-synuclein protein leads to white matter degeneration and neuronal injury. (2) Activated microglia. White matter damage and overexpression of α-synuclein activate microglia, resulting in the production of pro-inflammatory factors that cause neuronal injury and death. (3) Antigen presentation by activated microglia, leading to activation of CD4^+^ T cells and release of inflammatory cytokines such as IFN-γ, which exert cytotoxic effects on dopaminergic neurons. (4) Release of IL-1β, TNF-α, and IFN-γ by activated microglia promotes the transformation of A2 astrocytes into A1 astrocytes. (5) Under conditions of tissue ischemia and hypoxia, axons, and neuroglia in the white matter release various neurotransmitters, leading to neuronal deformation and death. (6) The abnormal aggregation of α-syn in oligodendrocytes simultaneously activates microglia, which release a large quantity of inflammatory mediators, promoting further abnormal activation of oligodendrocytes. This process leads to myelin defects and white matter damage, impacting neuronal function.

BBB disruption and increased permeability result in the ability of neurotoxic proteins such as β-amyloid (Aβ) and α-Syn, as well as T-cells, to have unrestricted access to the brain (Wu et al., 2021). In the brain tissue of patient with PD, misfolded α-Syn forms fibrillar aggregates, which not only cause damage within the neuronal cells, but may also affect the vascular system of the brain, particularly the BBB (Sui et al., 2014). The local inflammatory response and oxidative stress induced by these aggregates further disrupts the integrity of the BBB and increases its permeability. This increased permeability permits more inflammatory cells and other neurotoxic substances to cross the blood–brain barrier, causing neuronal damage, nerve fiber destruction and proliferation of some glial cells, which in turn damages the brain white matter (Figure 1). This process highlights the critical role of neurotoxic proteins in neurodegenerative diseases, particularly in the destruction of the BBB and disease progression.

### 2.2 Parkinson’s disease and white matter damage

In the brain tissue of patient with PD, white matter damage may reduce the efficiency of neural signal transmission, affecting communication between different brain regions and exacerbating both motor and non-motor symptoms (Zhu et al., 2023). White matter damage can significantly impact the immune environment of the CNS through various mechanisms, especially regarding the migration and function of microglia and peripheral immune cells (such as T cells, B cells, and macrophages; Stephenson et al., 2018). This alteration may lead to the accumulation of immune cells in the damaged areas, releasing large quantities of inflammatory mediators, further damaging nerve cells (Stephenson et al., 2018). Following white matter damage, microglia quickly activate and shift to a pro-inflammatory state, releasing cytokines and chemokines like TNF-α, IL-1β, and IL-6, which attract more immune cells to the damaged area and promote inflammation (Kou and VandeVord, 2014; Zhang et al., 2020). White matter damage may also impair BBB functionality (Toyota et al., 2019), allowing more peripheral immune cells, such as T cells and monocytes, and inflammatory factors to enter the brain, activating microglia and intensifying neuroinflammation.

Axons and glial cells within white matter can produce and release various important neurotransmitters, including but not limited to glutamate, GABA, glycine, adrenaline, acetylcholine, and dopamine (Butt et al., 2014). These neurotransmitters play key roles in the brain, not only in transmitting neural signals but also in regulating immune system functions. Notably, white matter damage might also disrupt these neurotransmitter systems, including the dopamine system, which is particularly important for PD patients. This alteration in neurotransmitter systems could affect patients’ motor functions and, through certain neurotransmitters like dopamine and norepinephrine, influence the activity of immune cells. Thus, white matter damage could disrupt the homeostasis of neurotransmitter systems, leading to the neurodegenerative characteristics of PD. Normally, certain components of the immune system can promote the repair and regeneration of nerve cells under proper regulation, helping to maintain the health of the nervous system. However, a persistent inflammatory environment may interfere with these neuroprotective responses, exacerbating the neurodegenerative process in the brain tissue of patient with PD. For example, studies in PD patients and animal models with ongoing inflammatory neurodegenerative processes show that peripheral inflammatory stimuli exacerbate neurodegeneration, highlighting the importance of controlling inflammation triggered by white matter damage and considering immune response modulation as a crucial strategy to protect the nervous system from further damage (Hasegawa et al., 2000; Pott Godoy et al., 2008, 2010).

Recent studies have underscored the importance of abnormalities in PD brain white matter structure and function, particularly changes in myelin and the role of oligodendrocytes (Yang et al., 2023). α-Syn, primarily associated with neurons, can also accumulate in oligodendrocytes (Gregorio et al., 2024). This not only affects the function of oligodendrocytes but also impacts myelin maintenance and neural signal transmission. Furthermore, oligodendrocytes might be compromised in an inflammatory environment, leading to myelin defects, and compromised white matter function, further affecting neural signal transmission (Yang et al., 2023). Specifically, neural fibers in the PD brain might suffer myelin damage due to oligodendrocyte dysfunction, leading to slowed neural signal transmission and affecting patients’ motor abilities and other neurological functions. As research into PD deepens, the role of oligodendrocytes and their associated myelin in the disease is increasingly recognized, yet studies on how oligodendrocytes promote PD pathological progression and the specific mechanisms and signaling pathways are scarce (Mavroeidi et al., 2022; Wang et al., 2022; Gregorio et al., 2024). Future research might reveal more about these cells’ specific mechanisms in PD, offering new directions for PD treatment.

## 3 Crosstalk between PD immune cells and white matter damage

Inflammation and shifts in immunity are pivotal characteristics in the development of PD, entailing interactions among various immune cells, inflammatory factors, chemokines, and other molecules. These changes extend beyond the CNS, incorporating the peripheral immune system as well. Among the earliest evidence of the immune system’s role in PD’s pathogenesis was the finding by McGeer et al. of activated microglia in the substantia nigra pars compacta during autopsies of PD patients (McGeer et al., 1988), where degeneration of dopaminergic neurons was also notably severe, suggesting a link between microglial activation and the disease’s mechanisms.

Substantial evidence suggests that both innate and adaptive immunity play crucial roles in the onset and progression of PD. Croisier et al. (2005), using CD68 and human leukocyte antigen-DR (HLA-DR) staining, further confirmed that microglial activation is directly related to the α-Syn load in PD patients’ brain tissues. α-syn, a key pathological feature of PD whose aggregates form Lewy bodies, indicates that α-syn can directly activate the innate immune system, sparking inflammatory responses. DAergic areas in the brain are regions rich in dopamine neurons crucial for regulating movement, reward, motivation, and emotions, with dysfunction associated with PD. The discovery of immune reactions in the substantia nigra striatum DAergic areas in PD patients supports the notion that immune dysfunction is involved in the disease’s etiology. Damaged cellular and humoral immune responses, the activation of inflammatory cells, and immune dysregulation are becoming recognized as pathological hallmarks of PD. Notably, the activation of brain microglia, the infiltration of peripheral T cells, and their interactions with microglia are significant. The disruption of these processes not only fosters an inflammatory environment but could also lead to the damage and death of neurons associated with PD. Regarding the link between white matter damage and the immune system, such damage could lead to BBB dysfunction, enabling peripheral immune cells (T cells and monocytes) and inflammatory factors to enter the brain. This not only intensifies inflammation within the central nervous system but might also activate more microglia, creating a pro-inflammatory vicious cycle, thus contributing to the inflammation and immune shifts within the PD brain. Moreover, recent studies have indicated the interaction between the brain’s lymphatic pathways and antigen-presenting cells, deepening the impact of the immune system on the PD process (Louveau et al., 2018; Brioschi and Colonna, 2019). This finding further validates the participation of innate and adaptive immune responses within the central nervous system in neurodegenerative diseases, revealing the complex interactions between white matter damage, the immune system, and PD.

Additionally, white matter damage plays a key role in the alterations of the immune microenvironment in the brain tissue of patient with PD, not only directly affecting the nervous system’s function but also by exacerbating inflammatory responses through the activation and migration of immune cells, thereby complicating the pathological progression of PD (Chiang et al., 2017). This underscores the necessity in PD treatment research to consider comprehensively the interactions between white matter damage, immune responses, and neurodegenerative changes, potentially offering significant insights for developing new therapeutic strategies. It also identifies possible targets for new treatment strategies for PD, especially those aimed at modulating immune responses, restoring BBB function, and preserving white matter integrity.

### 3.1 Microglial activation

Microglia are the resident immune cells of the brain, acting as the initial responders of the central nervous system’s innate immunity. They play crucial roles in monitoring the cellular environment, immune phagocytosis, antigen processing and presentation, participating in neuronal repair, remodeling, secreting neurotrophic factors, and chemokines (Katsumoto et al., 2014; Le et al., 2016). Microglia are densely distributed in the substantia nigra pars compacta and the striatum, both areas affected by PD(Wang et al., 2022). Research by Ouchi et al. (2005) using 11C-PK11195 imaging found increased activation of microglia in the brains of PD patients, especially in key areas affected by PD such as the striatum. This activation is directly related to the severity of the disease, indicating that the level of microglial activation can reflect the progression of PD. Another study showed that pro-inflammatory cytokines and chemokines expression, particularly TNF-α, IL-1β, IL-6, and IFN-γ, was upregulated in the brain tissue and cerebrospinal fluid of PD patients upon autopsy (Mogi et al., 1994a,b). Activated microglia are partly responsible for increased levels of TNF-α, IL-1β, TGFβ, IL-6, nitric oxide, and pro-apoptotic proteins in the substantia nigra pars compacta, striatum, and cerebrospinal fluid of PD patients (Nagatsu et al., 2000; Harms et al., 2021).

Furthermore, microglia, through their surface pattern recognition receptors like Toll-like receptors and NOD-like receptors, recognize and respond to abnormally aggregated α-syn, transitioning from a resting to an activated state, and releasing inflammatory mediators. This activation process, facilitated through specific pathways such as TLR4 and TLR2, and further through the NLRP3 inflammasome, promotes inflammatory responses (Fellner et al., 2013; Doorn et al., 2014; Duffy et al., 2018). Activated microglia have a toxic effect on dopaminergic neurons, can facilitate the spread of α-syn, activate T cell immune responses against neurons, and produce pro-inflammatory factors leading to neuronal damage (Govindarajan et al., 2020). Therefore, inhibiting microglial activation could help prevent the development of neurodegenerative lesions.

Regarding the association between white matter damage and microglia, excessive activation of microglia in PD leads to the release of a large amount of inflammatory mediators. The increase in inflammatory mediators may directly damage oligodendrocytes or interfere with their normal functions, affecting myelin formation and maintenance, thereby impacting the structure and function of white matter (Chiang et al., 2017). However, damage or loss of myelin can slow down or inaccurately transmit electrical signals through nerve fibers, affecting neuronal function and survival (Depp et al., 2023). On the other hand, the state of oxidative stress present in PD patients exacerbates damage to microglia and oligodendrocytes, leading to lipid peroxidation of cell membranes, protein oxidation, and DNA damage, further promoting the aggregation of α-syn and neurodegenerative lesions. For instance, some studies have shown that oligodendrocyte defects can cause aggregation of α-synuclein, axonal defects, activation of microglia, and astrocyte proliferation, revealing their role in neurodegenerative diseases and the complex pathological processes they cause (Festa et al., 2024; Gregorio et al., 2024).

In summary, the excessive activation of microglia in PD and the associated inflammatory responses, oxidative stress, and impact on oligodendrocytes collectively drive the progression of PD’s neurodegenerative lesions, providing important targets for PD treatment research.

### 3.2 Astrocytes in the brain tissue of patient with PD

Astrocytes play a complex role in the pathology of PD, offering both neuroprotective functions and potentially exacerbating neural damage (Yun et al., 2018). These cells regulate the brain environment through various means, including the release of neurotrophic factors, participation in neurotransmitter cycling, maintenance of ion balance, and support of the BBB (Volterra and Meldolesi, 2005; Abbott et al., 2006; Alvarez et al., 2013). However, in PD, the function of astrocytes may alter, shifting from protecting neurons to damaging them (Yun et al., 2018). Specifically, these cells can be categorized into two subtypes based on their reactivity: A1 and A2. A1 astrocytes produce pro-inflammatory mediators such as IL-1α, TNF-α, and C1q, are neuroinflammatory, can damage neurons and oligodendrocytes *in vitro*, exacerbate white matter lesions, induce apoptosis, and inhibit T cell activation and function. Conversely, A2 astrocytes have neuroprotective roles, promoting neuronal growth, survival, and synaptic repair (Ng and Ng, 2022; Lawrence et al., 2023).

During PD pathology, the activation of A1 astrocytes is toxic to neurons. Originally neuroprotective, under the influence of microglia in PD, they transform into the neurotoxic A1 subtype (Liddelow et al., 2017; Liddelow and Barres, 2017; Vilalta and Brown, 2018). For example, in the MPTP-induced mouse model, astrocytes lose their neuron-protective capacity and instead generate neurotoxicity upon encountering inflammatory cytokines like IL-1 and TNF-α (Liddelow et al., 2017). These A1 astrocytes exacerbate neuronal damage by releasing substances such as TNF-α (Tufekci et al., 2012). Moreover, dysfunction in astrocytes regarding α-Syn processing leads to its accumulation (di Domenico et al., 2019), further aggravating inflammation and oxidative stress, disrupting normal glutamate uptake and BBB function, and ultimately promoting neuronal death through excitotoxicity (Gu et al., 2010; Lee et al., 2010).

In PD patient’s brain, the pathological activation of A1 astrocytes not only directly impacts the health of white matter by promoting inflammation and damaging neurons and their support structures but also exacerbates these processes through interactions with the immune system. For instance, A1 astrocytes can activate microglia by releasing pro-inflammatory factors, further enhancing the inflammatory response, leading to more neural damage and white matter lesions (Kwon and Koh, 2020). Additionally, changes in astrocyte reactivity may affect their ability to regulate immune cells, altering the function of immune cells in the progression of PD (Wang et al., 2021). In summary, abnormal activation of astrocytes in PD inflicts multifaceted damage on the brain. These cells contribute to increased inflammation and damage to neurons and their surrounding structures (Wang et al., 2023), directly harming white matter areas. They also activate microglia by releasing pro-inflammatory factors (Kwon and Koh, 2020), not only worsening inflammation but also leading to broader neural damage and further alterations in white matter. Furthermore, such changes in astrocytes could also interfere with their normal ability to regulate immune cells, thereby affecting the role of immune cells in PD. Therefore, regulating the activation state and function of astrocytes is expected to provide a new strategy for the treatment of PD.

### 3.3 Oligodendrocytes in the brain tissue of patient with PD

Oligodendrocytes are primarily responsible for the production and maintenance of myelin sheaths in the central nervous system and are important supporters of maintaining neuronal health and function (Han et al., 2022). These cells may play a key role in PD’s pathology. Damage or dysfunction of oligodendrocytes may lead to degeneration of myelin. Myelin is the protective layer that wraps around nerve fibers and facilitates the rapid transmission of electrical signals. Damage to myelin in patients with PD may exacerbate abnormalities in nerve signaling, thereby exacerbating motor dysfunction (Kalafatakis and Karagogeos, 2021; Bae et al., 2023).

Recent studies emphasize the potential link between ferroptosis in white matter damage and PD, particularly focusing on oligodendrocytes (Wang et al., 2022). Dysregulated iron metabolism within oligodendrocytes could lead to iron accumulation, increasing the risk of ferroptosis and thereby harming white matter health (Ge et al., 2022; Li et al., 2022). Excessive iron accumulation may induce oxidative stress and trigger ferroptosis, further exacerbating neuronal damage. Increasing evidence suggests this process may play a crucial role in PD progression, especially by damaging myelin and nerve fibers, worsening cognitive and motor symptoms in PD patients (Wu et al., 2021; Wang et al., 2022; Lu et al., 2023). On the other hand, ferroptosis not only directly causes neuronal damage but also indirectly exacerbates PD pathology by activating the immune system and enhancing inflammatory responses (White, 2023; Xing et al., 2023). White matter damage, particularly in areas rich in oligodendrocyte-formed myelin, further confirms the role of ferroptosis in disrupting neural communication integrity. Given that ferroptosis in PD and white matter damage is not limited to causing cell death but also includes indirectly promoting disease progression through affecting immune responses and inflammatory processes, exploring therapeutic strategies to control ferroptosis and regulate immune responses becomes particularly important. This approach could offer a new therapeutic perspective for these neurodegenerative diseases, alleviating the damage and inflammation triggered by ferroptosis, slowing disease progression, and improving patient quality of life.

### 3.4 Peripheral immune cell infiltration involvement (T cells)

In the pathology of PD, peripheral immune cells such as T cells and monocytes may cross the damaged BBB and enter the brain. The infiltration of these cells further enhances the central immune response, releasing more inflammatory mediators and exacerbating neural damage. Studies have found an infiltration of CD3^+^ T cells (including both CD4^+^ and CD8^+^ T cells) in the brains of PD patients, especially in the substantia nigra pars compacta, with quantities significantly higher than in healthy control groups (Brochard et al., 2009). This discovery reveals the infiltration of peripheral immune cells, particularly T cells, in the CNS of PD.

In PD patient’s brain, pro-inflammatory T cells such as Th1 and CD8^+^ T cells may exacerbate neural damage by releasing inflammatory cytokines, while anti-inflammatory T cells like Th2 and Tregs may help alleviate inflammation. Research by Sulzer et al. (2017) found that CD8^+^T cells in PD patients can specifically recognize α-syn fragments, suggesting a key role for T cells in the development of PD. Furthermore, an increase in T cell numbers in PD models further confirms their specific role in the neurodegenerative process (Kurkowska-Jastrzebska et al., 1999; Theodore et al., 2008).

In PD patients, localized damage to the BBB has been observed in areas such as the ventral midbrain, deep cortical gray matter, and white matter (Kortekaas et al., 2005). This damage allows T cells to cross the BBB and enter the brain during both acute and chronic inflammation periods, as well as during routine surveillance by the brain’s lymphatic system. The infiltrating CD4^+^ T cells in the substantia nigra exhibit pro-inflammatory properties, capable of activating microglia and promoting the degeneration of dopaminergic neurons (Brochard et al., 2009; Reynolds et al., 2010). It has been found that the numbers of CD4^+^ and CD8^+^ T cells are increased in the peripheral blood of PD patients (Hisanaga et al., 2001). This phenomenon correlates with the expression levels of dopamine receptors on specific T cell subgroups in PD patients, implying that these receptors on immune cells may play a significant role in the development of PD (Baba et al., 2005). Moreover, T cells in PD patients show increased TNF receptor expression and increased production of inflammatory cytokines (IFN-γ and TNF-α) by Th1 cells, even in the presence of Tregs (Cebrián et al., 2014). While the direct cytotoxic effects of Th1 cells have not been reported directly, the release of inflammatory cytokines like IFN-γ can have a cytotoxic effect on dopaminergic neurons (Cebrián et al., 2014)and promote microglia-mediated local inflammatory responses, further exacerbating white matter damage. Additional evidence suggests that human dopaminergic neurons can directly activate CD8^+^ T cells by expressing MHC I molecules, leading to CD8^+^ T cell-mediated cytotoxic attacks (Cebrián et al., 2014; Zhang et al., 2023). These findings collectively indicate that the increase in number and dysfunction of T cells, especially CD4^+^ and CD8^+^ cells, are closely related to the pathogenesis of PD, highlighting the core role of adaptive immunity in PD. This offers a new perspective for PD treatment, suggesting that regulating adaptive immune responses, particularly the activity or number of T cells, could become a new target for PD therapy.

## 4 Discussion and conclusion

This review explores the interplay between PD-related white matter damage and microglia, astrocytes, oligodendrocytes, and peripheral immune cells, revealing how they exacerbate the neuroinflammatory environment and neurodegenerative changes in PD by producing and responding to pro-inflammatory cytokines (Figure 1). Unique to this review are: (i) a focus on how white matter damage, the immune system, and neuroinflammatory factors interact to further exacerbate PD progression; (ii) a shift from primarily concentrating on the substantia nigra in the midbrain to considering other brain regions, especially the white matter, highlighting its potential significant role in PD development; (iii) emphasizing the impact of white matter damage on the central nervous system’s immune environment, deepening our comprehensive understanding of PD’s pathological mechanisms. This review opens new directions for researching PD treatments, suggesting possibilities for developing more effective treatment methods to reduce neuroinflammation, protect neurons, slow disease progression, and improve patient quality of life.

Additionally, in other neurodegenerative diseases such as Multiple System Atrophy (MSA), the characteristic symptoms include Parkinsonian syndrome, cerebellar ataxia, autonomic failure, and cortical spinal dysfunction. In the brains of MSA patients, the hallmark pathology involves the aggregation of misfolded α-syn within oligodendrocytes, leading to the formation of glial cytoplasmic inclusions (GCIs) and subsequent neuronal dysfunction and death. The degeneration and activation of oligodendrocytes play crucial roles in the pathogenesis of MSA, potentially inducing oxidative stress and neuroinflammatory responses, thereby exacerbating neuronal damage and death. Additionally, oligodendrocytes pathology may trigger demyelination and loss of neurons, further hastening disease progression. While MSA and PD are distinct neurodegenerative disorders, both involve abnormal protein deposition within the nervous system. In PD, although abnormal deposition of α-syn primarily occurs in dopaminergic neurons, recent research has revealed the significant involvement of oligodendrocytes. Aberrant activation of oligodendrocytes may lead to increased inflammatory responses, impairing the function of surrounding neurons, while their involvement in modulating the activity of substantia nigra dopaminergic neurons may influence the development and clinical presentation of PD. Moreover, oligodendrocytes play critical roles in maintaining the integrity of brain white matter structure and neuronal function, and their aberrant activation or damage may result in disruption of white matter architecture and loss of neuronal function, further exacerbating the extent and scope of white matter damage.

White matter injury plays a central role in the pathology of PD, directly related to the clinical symptoms and progression of the disease. Structural changes in the white matter regions of PD patients are closely associated with cognitive decline and motor dysfunction, highlighting its importance as a therapeutic target. By understanding the mechanisms of white matter injury, we can gain deeper insights into the pathology of PD and develop targeted treatment strategies. Additionally, imaging assessments of white matter can serve as biomarkers for monitoring disease progression, which is crucial for early diagnosis and monitoring treatment effectiveness. Consequently, treatment strategies targeting white matter injury, such as strengthening the integrity of the BBB and reducing neuroinflammation, are showing promising translational potential. On the other hand, controlling the entry of peripheral immune cells and preventing BBB leakage are also key strategies. To reduce the invasion of peripheral immune cells, specific anti-inflammatory drugs or immunomodulators can be used, which work by inhibiting the activity of inflammatory mediators to alleviate neuroinflammation and protect neurons. For example, certain biologics and small molecule drugs can specifically block the adhesion molecules on the surface of immune cells, preventing them from crossing the BBB. Furthermore, enhancing the integrity of the BBB is crucial for preventing cell infiltration and neuronal injury. The use of antioxidants, metal chelators, or drugs that improve microvascular health can strengthen the structure and function of the BBB (Gomes and Negrato, 2014; Lee et al., 2020; Gao et al., 2023). For instance, antioxidants like N-acetylcysteine (NAC) have been found to reduce oxidative stress, thereby enhancing BBB stability, and reducing inflammation (Fan et al., 2020). By integrating these methods, we can effectively manage immune intervention and neuroprotection in PD, bringing new hope to patients.

For future research directions, the following suggestions are made: (i) Further exploration into the specific mechanisms of these interactions and their roles in PD is needed. Specifically, more in-depth studies on the effects of white matter damage on the immune system and inflammatory responses, and how regulating these interactions can develop more effective treatment methods are necessary. (ii) Attention should also be given to the role of peripheral immune cells (especially T cells) in PD and their interactions with the central nervous system. These studies will help deepen our understanding of PD’s pathological mechanisms and offer more possibilities for future clinical treatments.

## Author contributions

WM: Writing – original draft, Writing – review & editing. LW: Writing – original draft, Writing – review & editing. YG: Writing – review & editing. YL: Writing – review & editing. HP: Writing – review & editing. QW: Writing – review & editing. YZ: Writing – original draft, Writing – review & editing.
